# Unregulated access to health-care services is associated with overutilization—lessons from Austria

**DOI:** 10.1093/eurpub/cku189

**Published:** 2014-11-23

**Authors:** Otto Pichlhöfer, Manfred Maier

**Affiliations:** Department for General Practice and Family Medicine, Medical University of Vienna, A-1090 Wien, Austria

## Abstract

The Austrian health-care system is characterized by free provider choice and uncontrolled access to all levels of care. Using primary data, the ECOHCARE study shows that hospitalization rates for the secondary and tertiary care levels in Austria are both 4.4 times higher than those reported from the USA using a similar methodology. At the same time, essential functions of the primary care sector are weak. We propose that regulating access to secondary and tertiary care and restricting free provider choice to the primary care level would both reverse over utilization and strengthen the primary care sector.

## Introduction

The Austrian health-care system is characterized by universal health coverage and free provider choice. At the same time, the functions of the primary care sector are weak when compared with countries with similar socioeconomic conditions. Especially, the performance of primary care in the areas of continuity of care and coordination of care is suboptimal.^1^^,^^2^ Austria ranked only 10th among 14 countries on a primary care development scale and thus was classified as a ‘low primary care’ country.^3^ Hospital admission rates are very high and the secondary and tertiary care levels suffer from overutilization.^4^

The Austrian health-care system is based on the Bismarck Model and is largely financed by sickness funds which draw on mandatory contributions from both employers and employees. Today, 99% of the Austrian population have health insurance. In 2010, total health spending accounted for 11% of Gross Domestic Product (GDP) in Austria while the Organisation for Economic Co-operation and Development (OECD) average was 9.5% (USA 17.6%). These costs were funded to 76.2% by public sources (OECD average 72.2%), of which 50% had to be financed by health insurance funds. In a classification of the OECD (2012) with regard to the factors LE at birth and at age 65 for males and females and infant mortality, Austria ranked in the same cluster with Denmark and the Netherlands. In Austria, the number of the total physician workforce is very high at 4.8 physicians per 1000 population (OECD average 3.1). On the other hand, Austria has only 7.7 nurses per 1000 population (OECD average 8.7). The number of curative care beds is among the highest found and stood at 5.5 per 1000 population (OECD average 3.4). Austrians enjoy above average life expectancy at birth; 80.7 years (OECD average 79.8).

In the prevailing public opinion, the guiding principles for health policy are universal and free health coverage and free and unrestricted provider choice for patients.

This ‘laissez-faire’ approach towards both free choice of provider and free access to any level of care has led to a situation where patients can virtually selfrefer themselves wherever they like.^1^^,^^4^ We want to demonstrate, that unregulated patient access to all levels of medical care is linked to several unwanted developments for the health-care system as a whole and in particular for essential primary care functions.

## Methods

In 2011, we conducted the trial ‘Ecology of Medical Care—Utilisation of Health Care in Austria’ (ECOHCARE; ClinicalTrials.gov Identifier: NCT01261845) in which we assessed the utilization of health care services by the Austrian population. The methods used and parts of the results for subgroups of patients from this study have been published.^5^ We chose a recall period of 1 month which enabled us to compare our results with the studies of Green (2001) and White (1961) in the USA who used a similar methodology.^6^ We will use this comparison to show certain characteristics of the Austrian system.

## Results

**Figure 1:**
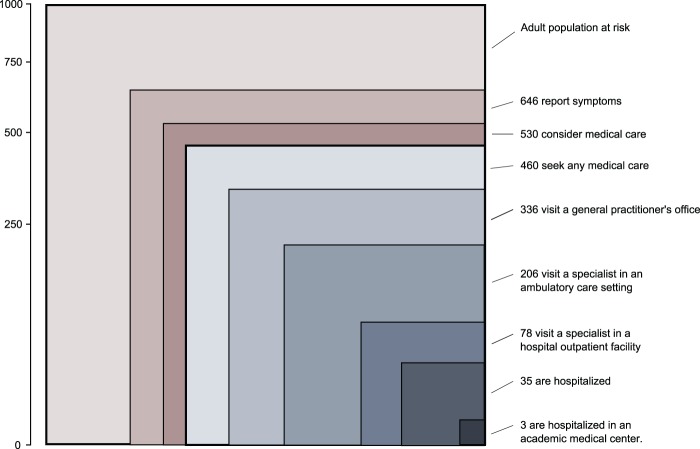
Monthly prevalence estimates of illness in the community and the utilisation of levels of care. Data are for persons 16 years of age and older. Each box represents a subgroup of the largest box, which comprises 1000 persons. The figure has been designed to allow comparison with Green.^6^

Among the 3500 Austrians (100%) interviewed who were 16 years of age or older, 64.6% (95% CI 62.2, 67.0) reported any kind of health complaint (figure 1). In our sample, 53.0% (95% CI 50.4, 55.5) reported that they had considered seeking medical care. This is 1.6 times the number of Green. Eventually, 46.0% (95% CI 43.5, 48.6) received some form of medical care (2.1 times Green). A primary care physician was consulted by 33.6% (95% CI 31.2, 36.1) (3.0 times Green) and 20.6% (95% CI 18.5, 22.6) visited a specialist physician (2.0 times Green). A hospital outpatient clinic was consulted by 7.8% (95% CI 6.4, 9.2; 2.3 times Green). Finally, 3.5% (95% CI 2.5, 4.6) were hospitalized in a secondary care institution (4.4 times Green) and 0.31% (95% CI 0.07, 0.56) were hospitalized in an academic medical centre (4.4 times Green). Similar results for accessing health services in Austria based on secondary data for analysis have recently been reported.^4^ Therefore these data are convincing.

## Discussion

The political interpretation of free provider choice in Austria implies unregulated patient access to all levels of care including for example through selfreferral. This has created a system with overall high utilization, especially prominent in the secondary and tertiary care sectors with utilization rates four times those found in the USA (see figure 1). Excessive numbers of patients attending university hospitals for routine care have created a burden on care structures and staff in tertiary care institutions which should focus to deliver sophisticated care for rare and complex diseases. The overutilization of hospital care is especially of concern because of the increase in hospital-care associated morbidity: in EU Member States, between 8 and 12% of patients admitted to hospitals suffer from adverse events.

In an Australian study, a hospital stay carried a 5.5% risk of an adverse drug reaction and a 17.6% risk of infection and each additional night in the hospital increased the risk by 0.5% for adverse drug reactions and 1.6% for infections.

In Austria, hospital costs are the highest within the OECD at 36% of total health-care costs. The magnitude of the economic problem of the overhospitalization can be illustrated by estimating that reducing the excess costs from the OECD average by half would decrease health expenditure by 2% of the GNP.

The Austrian example shows that interpreting the principle of free provider choice as unregulated access to all levels of care undermines several key features of a good health-care system: (i) Comprehensive care, meaning that all health problems in the population, with the exception of those too rarely encountered, should be dealt with in primary care, including the need of short-term referral. This includes the integration between primary and secondary care and the breaking down of the vertical silos of single-issue approaches^7^; (ii) coordination of care, meaning that the primary care practice must integrate all aspects of care when patients must be seen by another specialist^2^; (iii) equity in health, which includes working for the specific needs of subpopulations and protecting them from under- or overmedication and overhospitalization. Comprehensiveness and coordination cannot be achieved since patients consult specialists in an arbitrary way. Therefore medical histories are fragmented and disseminated at the various provider locations, which makes comprehensive access to information and therefore efficient coordination of care impossible. If patients’ health-care utilization is distributed across many specialities and health-care levels in an uncontrolled way, personalized care and equity is difficult to achieve.

In spite of these shortcomings a high proportion of the Austrian population (two thirds) expresses high self-reported patient satisfaction and rates the current state of the health-care system as ‘very good’ or ‘good’. In a European comparison, Austria thus ranks fourth in popularity among citizens behind Belgium, Luxembourg, and Finland.^1^ This can be partly attributed to the fact that the choice of provider lies entirely with the patients which gives them a sense of misguided empowerment.

However in a recent US study, higher patient satisfaction was associated with greater inpatient use and even increased mortality.^8^

Thus, uncontrolled access to all levels of care leads to high utilization of health services and is associated with weak primary care functions. Based on the evidence available, we propose that the implementation of policies that would regulate access to secondary and tertiary care and restrict free provider choice to the primary care level would reverse overutilization and strengthen the primary care sector in Austria and would facilitate the much needed link between public health and primary care. This is in line with a recent Dutch study that proposes patient panels or practice lists to strengthen the continuity of care and community orientation.^9^

Should these recommendations be implemented, the rate of self-reported patient satisfaction, which is not in line with other European countries when compared with life expectancy, could decrease but objective health measures should improve.^10^

We firmly believe that the Austrian health-care system and the primary care sector in particular, along with the population it serves, would greatly benefit by the proposed policy changes.
